# Revisiting the symptom iceberg in today's primary care: results from a UK population survey

**DOI:** 10.1186/1471-2296-12-16

**Published:** 2011-04-07

**Authors:** Alison M Elliott, Anne McAteer, Philip C Hannaford

**Affiliations:** 1 Senior Research Fellow, Centre of Academic Primary Care, Institute of Applied Health Sciences, University of Aberdeen, Foresterhill Health Centre, Westburn Road, Aberdeen, AB25 2AY, UK; 2 Research Assistant, Centre of Academic Primary Care, Institute of Applied Health Sciences, University of Aberdeen, Foresterhill Health Centre, Westburn Road, Aberdeen, AB25 2AY, UK; 3 Grampian Health Board Chair of Primary Care, Centre of Academic Primary Care, Institute of Applied Health Sciences, University of Aberdeen, Foresterhill Health Centre, Westburn Road, Aberdeen, AB25 2AY, UK

**Keywords:** Signs and symptoms, Symptom iceberg, Community-based, Health care services, Primary care

## Abstract

**Background:**

Recent changes in UK primary care have increased the range of services and healthcare professionals available for advice. Furthermore, the UK government has promoted greater use of both self-care and the wider primary care team for managing symptoms indicative of self-limiting illness. We do not know how the public has been responding to these strategies. The aim of this study was to describe the current use of different management strategies in the UK for a range of symptoms and identify the demographic, socio-economic and symptom characteristics associated with these different approaches.

**Methods:**

An age and sex stratified random sample of 8,000 adults (aged 18-60), drawn from twenty general practices across the UK, were sent a postal questionnaire. The questionnaire collected detailed information on 25 physical and psychological symptoms ranging from those usually indicative of minor illness to those which could be indicative of serious conditions. Information on symptom characteristics, actions taken to manage the symptoms and demographic/socio-economic details were also collected.

**Results:**

Just under half of all symptoms reported resulted in respondents doing nothing at all. Lay-care was used for 35% of symptoms and primary care health professionals were consulted for 12% of symptoms. OTC medicine use was the most common lay-care strategy (used for 25% of all symptom episodes). The GP was the most common health professional consulted (consulted for 8% of all symptom episodes) while use of other primary care health professionals was very small (each consulted for less than 2% of symptom episodes). The actions taken for individual symptoms varied substantially although some broad patterns emerged. Symptom characteristics (in particular severity, duration and interference with daily life) were more commonly associated with actions taken than demographic or socio-economic characteristics.

**Conclusion:**

While the use of lay-care was widespread, use of the primary care team other than the GP was low. Further research is needed to examine the public's knowledge and opinions of different primary care services to investigate why certain services are not being used to inform the future development of primary care services in the UK.

## Background

Symptoms are powerful drivers of healthcare utilisation. Although many symptoms are managed without the input of healthcare professionals, symptoms such as cough, headache and fatigue remain common reasons for seeking medical care [1,2]. The development in the UK of primary care teams (including practice nurses and community pharmacists) and new services (including nurse-led telephone advice lines and out-of-hours primary care services) has increased the range of healthcare professionals available for advice. Furthermore, UK government policy has advocated greater use of these services for the management of common ailments [3], and promoted self-care of symptoms indicative of self-limiting illness [4,5]. Similar policies are being implemented elsewhere and the World Health Organization has highlighted the increasing importance of self-care [6].

There have been relatively few community-based studies investigating the publics' responses to a range of different symptoms and none in the UK since these primary care changes were introduced. We therefore do not know how the public has been responding to recent strategies and we do not have a current picture of the UK symptom iceberg [7,8]. Specifically, we do not know for different symptoms, the size of the visible part of the iceberg (representing the proportion of symptoms presented to medical care) and the submerged part (representing the proportion which is not).

Whilst recent primary care changes are likely to benefit people, they could be detrimental if they prevent, or delay, individuals with symptoms of potentially serious disease from seeking healthcare, or if they encourage people to self-manage inappropriately. A clearer understanding of how people are currently managing their symptoms and what influences this management is therefore required. This paper describes current use of different management strategies in the UK for a range of symptoms and identifies factors associated with these different approaches.

## Methods

### Subjects and sampling

A UK-wide population-based postal survey was undertaken in 2007/2008. Full details of the methods have been published previously [9]. In brief, an age- and sex-stratified random sample of 8,000 adults aged 18-60 was drawn from 20 UK general practices following ethical approval. The sample size was calculated to ensure we could estimate the prevalence of the identified symptoms with narrow two-sided 99% confidence intervals around the prevalence estimates. Practices were recruited from the nationally representative Medical Research Council General Practice Research Framework. Convenience sampling was used to select practices that varied in size, geographical location, level of deprivation and area type. GPs screened the sample and excluded anyone who they felt it would be inappropriate to approach. Practice staff sent out questionnaire packs on our behalf. A reminder and replacement questionnaire was sent to non-respondents after three weeks.

### Questionnaire

The questionnaire inquired about the occurrence of 25 physical and psychological symptoms in the last two weeks. Symptoms were identified from previous literature and pilot work and ranged from those usually indicative of minor or self-limiting illness through to those which could be indicative of potentially serious conditions. A two week time period was chosen as this was considered long enough to enable many symptoms to have lasted their full course and for actions to have been taken, but was short enough to ensure good recall. For each symptom experienced, respondents indicated: the severity of the symptom at its worst; how long it had lasted; how much it had interfered with daily life; and how often they had experienced it in the previous year. Respondents were also asked to indicate all actions they had taken in the last two weeks to manage their symptom(s) from: did nothing; looked for information; discussed with friends and family; consulted a GP; phoned NHS 24/NHS Direct (a telephone advice line available 24 hours a day in the UK which co-ordinates all out-of-hours primary care services); consulted a nurse; consulted a pharmacist; consulted a complementary therapist; took over-the-counter (OTC) medications; took prescribed medications; other, please specify. Comprehensive data were also collected on respondent characteristics including: gender, age, marital status, social support, education, housing, employment, household income, ethnicity, smoking, and the presence of a chronic condition.

### Symptom seriousness

A random sample of 30 GPs from Grampian, Scotland were sent a self-completion postal questionnaire asking them to rate the potential seriousness of each of the 25 symptoms being examined as either:

• A - a symptom not causing concern, usually indicative of trivial/self-limiting illness

• B - a symptom of moderate concern - neither trivial nor serious

• C - a symptom of concern that could be indicative of a serious condition or illness

Thirteen GPs (43%) completed the ratings. From their responses a simple five-level symptom seriousness index was developed: Level 1 (least serious) - most GPs rated the symptom as A, Level 2 - GPs rated the symptom as a mix of As and Bs, Level 3 - most GPs rated the symptom as B, Level 4 - GPs rated the symptom as a mix of Bs and Cs, Level 5 (most serious) - most GPs rated the symptom as C. Examination of agreement between the GPs found that 81% of the GPs rated the symptom at the level it was assigned to, 18% rated it at one level higher or lower than it was assigned to and 1% rated it at two levels higher or lower than it was assigned to. The symptoms assigned to each of the five categories are shown in column 1 of Table 1.

**Table 1:** Actions taken for symptoms experienced in the last two weeks (proportion of symptoms)*

Level of seriousness based on GP ratings	Symptoms	n^	Did nothing at all †	Looked for information	Discussed with friends & family	Took over-the-counter medicines	Phoned NHS24/NHS Direct	Consulted nurse	Consulted pharmacist	Consulted comple-mentary therapist	Consulted GP (on phone or in person)	Took prescribed medicines
1	Feeling tired/run down	887	69.9	3.0	13.6	3.5	0.3	0.8	0.7	1.4	6.8	4.4
	Difficulty sleeping	607	64.3	3.1	11.9	7.9	0.5	0.3	1.0	1.3	6.8	7.4
	Sore throat	411	44.0	1.9	9.0	43.3	1.0	1.2	4.6	0.7	7.3	5.4
	Cold or flu symptoms	372	38.4	1.6	9.4	51.6	0.8	1.9	4.3	0.5	6.7	4.0
	Diarrhoea	266	60.9	1.5	5.6	17.3	0.4	0.8	1.9	0	7.1	10.2
	Loss of appetite	117	74.4	4.3	12.8	1.7	0.9	0	0.9	0	10.3	3.4

2	Back pain	653	40.9	2.5	7.0	29.7	0.2	0.8	0.3	6.6	6.0	13.6
	Nervousness/anxiety	405	55.3	3.2	18.0	5.4	0.2	1.0	0.7	3.0	10.9	11.1
	Cough	372	52.7	1.6	7.8	31.7	0.3	1.1	6.2	0.5	7.0	6.2
	Nausea/feeling sick	259	68.3	1.5	8.5	8.9	0.8	0.4	1.5	0	8.5	10.0
	Constipation	203	46.8	3.9	5.9	24.1	0.5	1.0	2.5	0.5	4.4	15.3
	Vomiting	95	63.2	1.1	9.5	10.5	1.1	2.1	3.2	0	13.7	6.3

3	Headaches	845	23.0	1.1	4.3	65.7	0.4	0.1	1.8	0.7	2.0	7.6
	Joint pain	678	35.4	4.6	10.3	28.9	0.3	0.7	1.0	4.7	11.4	17.7
	Indigestion/heartburn	392	29.6	2.0	6.1	51.0	0.3	0.3	2.8	1.0	5.6	14.3
	Feeling depressed	353	53.0	4.0	17.8	4.0	0.3	2.0	0.3	2.3	13.3	15.9
	Stomach/abdominal pain	337	42.7	3.3	10.4	24.0	0.9	1.2	0.9	1.5	13.9	19.3
	Dizziness	194	69.1	1.5	8.2	2.6	0.5	1.0	1.0	0	8.8	9.3
	Wheezy chest	158	41.1	1.9	5.1	11.4	1.3	1.3	2.5	0.6	16.5	33.5
	Fainting	14	64.3	7.1	7.1	0	0	0	0	0	14.3	7.1

4	Shortness of breath	176	47.7	2.3	9.1	4.5	1.1	0.6	1.7	0	18.2	31.8
	Blood in stool	52	57.7	5.8	11.5	5.8	0	1.9	1.9	0	23.1	7.7
	Unintentional weight loss	37	59.5	5.4	16.2	2.7	2.7	0	5.4	0	27.0	5.4

5	Chest pain	108	52.8	1.9	13.0	4.6	1.9	0.9	1.9	0.9	15.7	19.4
	Coughing up blood	4	75.0	0	0	0	0	0	0	0	0	25.0

	**ALL SYMPTOMS**	**7994**	**48.6**	**2.6**	**9.8**	**25.0**	**0.5**	**0.8**	**1.8**	**1.8**	**8.3**	**11.1**

* Respondents could tick as many boxes as they wanted to reflect that they may have taken more than one action for their symptoms in the last two weeks

^ For each of the 25 individual symptoms n refers to the number of different individuals reporting the symptom in the previous two weeks. For all symptoms combined n refers to the total number of symptoms experienced in the last two weeks (since people reported multiple symptoms the n does not refer to the number of individuals)

† Refers to individuals who ticked the 'did nothing' option and ticked no other actions

### Analysis

To minimize the chances of a type 1 error from multiple testing, a conservative p-value of < 0.01 was used to denote statistical significance. Basic descriptive analyses were used to examine the actions taken for all symptoms combined and for individual symptoms. Binary logistic regression was used to examine:

1. factors associated with each individual action taken for all symptoms combined.

2. factors associated with a) the use of lay-care (looked for information, discussed with friends and family, took OTC medications and/or reported the use of other lay-care strategies) and b) the use of primary care health professionals (consulted a GP, nurse, pharmacist, complementary therapist and/or phoned NHS24/NHS Direct) for individual symptoms.

The first analysis was undertaken to explore the factors associated with each of the individual actions. The second analysis was undertaken to allow the effect of individual symptom characteristics such as severity to be investigated and because combining symptoms can mask significant variations [10-13]. In the second analysis the actions taken had to be aggregated due to small numbers and only the 12 most prevalent symptoms could be examined.

Unadjusted and adjusted odds ratios and 99% confidence intervals were calculated. For consistency across the models, odds ratios were adjusted for all other variables being tested. In analysis 1, odds ratios were adjusted for all participant characteristics (gender, age, marital status, social support, education, housing, employment, household income, ethnicity, smoking, presence of a chronic condition) and number of symptoms experienced in the last two weeks. In analysis 2, odds ratios were adjusted for all participant characteristics and four symptom characteristics (severity, duration, interference with life and recent experience). For ease of reporting, responses to questions about symptom characteristics were categorised as: 'not severe' (mild, tolerable or moderate) and 'severe' (severe or extremely severe); 'short duration' (<1 day, 1-2 days, 3-4 days, 5-6 days), 'medium duration' (1-4 weeks) and 'long duration' (>4 weeks); 'low interference' (not at all, slightly or moderately) and 'high interference' (quite a bit or extremely); 'no recent experience' (had not experienced the same symptom in the previous year) and 'recent experience' (had experienced the same symptom in the previous year).

## Results

Full details of the response rate, participant characteristics and symptom prevalence have been published previously [9]. A total of 46.4% of questionnaires were returned, of which 2,474 had complete data and were included in the analyses giving a corrected completed response rate of 33.2%. Most demographic and socio-economic groups (except non-whites) were well represented in the sample.

### Actions taken in the last two weeks

For almost half of all symptoms reported respondents chose to do nothing at all over the two week period. Thirty-five per cent of symptoms resulted in the use of lay-care, usually OTC medicine use (25%). Twelve per cent of symptoms led to a consultation with a primary care health professional, usually the GP (8%). Prescription medicines were used for just over 10% of symptoms.

There was considerable variation in the actions taken for individual symptoms (Table 1), although some broad management patterns emerged. The proportion of people seeking information about their symptom was highest for more serious symptoms such as *fainting *and *blood in stool*. The proportion of respondents who discussed their symptom with family and friends was highest for psychological symptoms (*nervousness/anxiety *and *feeling depressed*) and more serious symptoms (*unintentional weight loss *and *chest pain*). The proportion of people using an OTC medicine was highest for minor symptoms (*headaches *and *cold or flu symptoms*).

The proportion of people using NHS 24/NHS Direct was higher for more serious symptoms (*unintentional weight loss *and *chest pain*), although even then the levels of usage remained low (<3%). Few respondents consulted a nurse for their symptoms and there was no clear pattern in the type of symptoms presented. Slightly more respondents consulted a pharmacist, usually for less serious symptoms such as *cough *and *sore throat*. *Back pain *and *joint pain *were the symptoms most commonly presented to a complementary therapist. The GP was the primary care health professional consulted most often, especially for serious symptoms (*unintentional weight loss *and *blood in stool*) and respiratory symptoms (*shortness of breath *and *wheezy chest*). Respiratory symptoms (*shortness of breath *and *wheezy chest*) were the symptoms most likely to result in the use of a prescription medicine. There was no clear pattern in which symptoms people chose to do nothing about.

### Factors associated with actions taken

Factors associated with each of the actions taken varied considerably (Table 2). For example: women and those with at least three symptoms were more likely than men or those with 1-2 symptoms respectively to have used an OTC medicine; those with an annual household income of £30,000 or more were less likely than those with an annual household income of less than £15,000 to have consulted a nurse; those no longer married, those with a chronic condition and those with six or more symptoms were all more likely to have consulted a GP than those in the reference group for each variable. The number of symptoms people experienced was the factor most commonly associated with the action taken, with those experiencing a higher number of symptoms being more likely than those experiencing 1-2 symptoms to take some form of action (with significant associations for lay-care actions, consultation with the GP and use of prescription medicines).

**Table 2:** Associations between actions taken for all symptoms in the last two weeks and participant characteristics and number of symptoms (unadjusted and adjusted odds ratios)

Participant characteristics and number of symptoms	Looked for information		Discussed with friends/family		Took OTC medicines		Phoned NHS 24/NHS Direct		Consulted nurse		Consulted pharmacist		Consulted comp therapist		Consulted GP		Took prescribed medicines	
	**UOR**	**AOR**	**UOR**	**AOR**	**UOR**	**AOR**	**UOR**	**AOR**	**UOR**	**AOR**	**UOR**	**AOR**	**UOR**	**AOR**	**UOR**	**AOR**	**UOR**	**AOR**

**Gender**																		
Men ^†^	1.00	1.00	1.00	1.00	1.00	1.00	1.00	1.00	1.00	1.00	1.00	1.00	1.00	1.00	1.00	1.00	1.00	1.00
Women	0.80	0.75	1.23	1.11	**1.63**	**1.46**	0.73	0.64	1.82	1.44	1.51	1.36	**2.79**	**2.71**	1.25	1.22	1.29	1.40

**Age**																		
18-24 ^†^	1.00	1.00	1.00	1.00	1.00	1.00	1.00	1.00	1.00	1.00	1.00	1.00	1.00	1.00	1.00	1.00	1.00	1.00
25-34 yrs	0.86	0.82	0.60	0.67	1.63	1.66	0.87	1.56	0.28	0.40	0.51	0.79	0.69	0.64	0.70	0.54	1.35	1.13
35-44 yrs	0.98	1.04	**0.50**	0.62	1.29	1.44	1.01	1.26	0.84	0.88	0.63	1.12	1.37	1.57	0.89	0.64	1.08	0.73
45-54 yrs	0.86	0.98	**0.43**	0.57	1.15	1.44	0.97	0.82	0.80	0.77	0.41	0.58	1.93	2.19	1.06	0.68	**1.99**	1.15
55-60 yrs	1.37	1.23	**0.32**	**0.39**	1.04	1.26	0.62	0.42	0.62	0.19	0.36	0.39	1.89	2.19	1.22	0.60	**2.50**	1.20

**Marital status**																		
Single ^†^	1.00	1.00	1.00	1.00	1.00	1.00	1.00	1.00	1.00	1.00	1.00	1.00	1.00	1.00	1.00	1.00	1.00	1.00
Married/living together	0.94	0.77	0.73	1.35	1.05	1.04	0.79	1.58	0.81	2.96	0.72	1.36	1.19	0.82	1.15	1.48	1.17	1.31
No longer married	0.99	0.87	0.76	1.23	1.14	0.97	1.43	2.31	1.93	2.99	0.95	1.47	1.97	1.04	**2.75**	2.80	1.48	0.71

**Social support**																		
Low ^†^	1.00	1.00	1.00	1.00	1.00	1.00	1.00	1.00	1.00	1.00	1.00	1.00	1.00	1.00	1.00	1.00	1.00	1.00
Medium	1.81	2.52	0.73	0.74	1.42	1.45	0.37	0.35	0.33	0.31	0.67	0.67	0.95	0.83	0.61	0.59	0.57	0.91
High	1.54	1.94	0.88	0.89	1.30	1.25	**0.11**	**0.10**	0.26	0.27	1.03	1.15	0.85	0.76	0.61	0.76	**0.49**	0.93

**Education**																		
No qualifications ^†^	1.00	1.00	1.00	1.00	1.00	1.00	1.00	1.00	1.00	1.00	1.00	1.00	1.00	1.00	1.00	1.00	1.00	1.00
Secondary school	1.06	1.39	1.56	1.91	1.47	1.50	1.45	3.23	0.96	1.31	0.88	0.67	1.55	3.01	0.70	1.03	**0.48**	0.92
Higher education	1.31	1.56	1.60	2.10	1.31	1.43	0.70	1.65	0.58	1.16	0.52	0.38	1.51	3.04	**0.46**	0.69	**0.36**	0.77

**Housing**																		
Owned/mortgaged ^†^	1.00	1.00	1.00	1.00	1.00	1.00	1.00	1.00	1.00	1.00	1.00	1.00	1.00	1.00	1.00	1.00	1.00	1.00
Privately rented and other	0.63	0.61	1.20	0.92	0.93	0.84	1.06	0.55	1.19	0.32	1.37	1.01	1.08	1.55	0.94	0.59	0.95	0.73
Council/housing assoc.	0.56	0.56	1.25	1.11	0.83	0.73	2.32	1.79	0.42	-	2.26	1.58	1.02	1.40	1.52	0.80	1.55	0.61

**Employment**																		
Full-time ^†^	1.00	1.00	1.00	1.00	1.00	1.00	1.00	1.00	1.00	1.00	1.00	1.00	1.00	1.00	1.00	1.00	1.00	1.00
Part-time	1.00	0.79	0.85	0.84	1.12	0.95	0.61	1.06	0.69	0.29	1.34	1.26	1.68	1.40	1.00	0.81	1.21	0.99
Self-employed	1.01	0.98	0.72	0.78	0.85	0.75	0.70	1.07	1.06	0.85	1.32	1.25	1.62	1.78	0.57	0.55	0.80	0.56
Not working due to illness	1.00	0.68	0.98	0.56	0.59	0.41	2.18	0.86	3.33	0.90	2.08	1.27	1.54	1.25	**3.21**	1.40	**11.16**	**5.63**
Others not in employment	0.91	0.86	1.39	1.29	0.99	0.87	1.99	2.00	2.78	1.85	1.78	1.25	1.71	1.36	0.86	0.65	1.19	0.79

**Income**																		
<£15,000 ^†^	1.00	1.00	1.00	1.00	1.00	1.00	1.00	1.00	1.00	1.00	1.00	1.00	1.00	1.00	1.00	1.00	1.00	1.00
£15,000-29,999	0.91	0.75	0.79	0.74	0.97	0.78	0.54	0.67	0.43	0.28	0.49	0.64	0.95	1.07	0.69	0.83	0.71	0.87
£30,000-49,000	1.03	0.95	0.78	0.73	0.87	0.69	1.26	1.71	0.33	**0.18**	0.68	0.82	0.71	0.96	**0.49**	0.68	**0.54**	0.82
£50,000+	0.66	0.56	0.68	0.60	0.84	0.65	0.81	1.54	**0.13**	**0.08**	0.41	0.67	0.66	0.88	**0.48**	0.82	**0.34**	0.55

**Ethnicity**																		
White ^†^	1.00	1.00	1.00	1.00	1.00	1.00	1.00	1.00	1.00	1.00	1.00	1.00	1.00	1.00	1.00	1.00	1.00	1.00
Other	0.31	0.37	**2.47**	2.38	1.02	0.96	1.89	-	2.44	-	**4.58**	**5.30**	0.44	0.50	1.04	1.26	1.04	1.00

**Smoking**																		
Never ^†^	1.00	1.00	1.00	1.00	1.00	1.00	1.00	1.00	1.00	1.00	1.00	1.00	1.00	1.00	1.00	1.00	1.00	1.00
Ex-smoker	1.17	0.88	0.93	1.01	0.96	0.93	3.27	2.74	1.82	1.18	1.00	1.06	1.18	1.21	1.33	1.17	**1.62**	1.36
Current smoker	0.67	0.60	1.11	0.91	0.81	0.75	1.25	0.52	0.97	0.80	1.40	0.87	0.62	0.60	1.41	0.94	1.32	0.89

**Chronic condition**																		
No ^†^	1.00	1.00	1.00	1.00	1.00	1.00	1.00	1.00	1.00	1.00	1.00	1.00	1.00	1.00	1.00	1.00	1.00	1.00
Yes	1.32	1.16	0.98	0.90	1.08	1.04	1.57	0.77	1.35	1.06	1.08	1.11	1.25	1.00	**2.38**	1.66	**5.28**	**3.78**

**Number of symptoms**																		
1-2 ^†^	1.00	1.00	1.00	1.00	1.00	1.00	1.00	1.00	1.00	1.00	1.00	1.00	1.00	1.00	1.00	1.00	1.00	1.00
3-5	1.38	1.57	**1.62**	**1.77**	**2.04**	**2.09**	1.70	1.54	1.19	1.44	2.16	2.35	1.33	1.37	**1.95**	1.67	**2.61**	**2.09**
6-9	**2.33**	**2.72**	**2.27**	**2.38**	**2.87**	**2.92**	4.06	3.47	3.32	3.23	**2.81**	2.45	1.37	1.35	**3.66**	2.41	**4.30**	**2.66**
10+	1.40	2.06	**2.52**	**2.90**	**2.66**	**2.94**	7.86	7.96	**6.39**	5.93	1.87	1.74	1.85	2.81	**7.45**	4.68	**9.15**	**4.68**

^† ^Referent group

UOR - Unadjusted odds ratio

AOR - Adjusted odds ratio (adjusted for all variables, except when the variable itself was being examined)

Figures highlighted in bold are significant at 1% level (p < 0.01) (99% CIs are not presented to simplify presentation)

- missing data due to small numbers for some of the actions and sub-groups being examined

Table 3 shows the factors associated with lay-care for six selected symptoms (chosen to reflect physical, psychological, acute, chronic, minor and moderate symptoms). Few participant characteristics were associated with the use of lay-care for these symptoms. Women, those with high social support and non-whites were all more likely to use lay-care than those in the reference group for each of these characteristics, although the associations were only statistically significant in one or two symptoms. Symptom characteristics (i.e. high severity, longer duration, and high interference with life) were more often associated with the use of lay-care than personal characteristics. Although many of the associations lost their statistical significance after adjustment, the trends remained.

**Table 3:** Associations between use of lay-care for selected symptoms and participant and symptom characteristics (unadjusted and adjusted odds ratios)

Participant and symptom characteristics	Feeling tired/ run down		Cold or flu symptoms		Back pain		Nervousness/ anxiety		Headaches		Joint pain	
	
	UOR	AOR	UOR	AOR	UOR	AOR	UOR	AOR	UOR	AOR	UOR	AOR
**Gender**												
Men ^†^	1.00	1.00	1.00	1.00	1.00	1.00	1.00	1.00	1.00	1.00	1.00	1.00
Women	1.30	1.29	1.74	1.64	**1.66**	1.32	0.95	0.74	**1.99**	**2.11**	1.32	1.24

**Age**												
18-24 ^†^	1.00	1.00	1.00	1.00	1.00	1.00	1.00	1.00	1.00	1.00	1.00	1.00
25-34 yrs	0.74	0.61	0.91	0.84	1.68	3.64	0.67	0.87	1.71	1.51	1.21	4.01
35-44 yrs	0.91	1.01	1.14	1.36	1.94	3.17	0.46	0.99	2.42	2.62	1.18	4.66
45-54 yrs	0.97	1.14	1.24	2.20	1.19	3.30	0.58	0.86	1.98	2.33	1.32	5.04
55-60 yrs	0.76	0.70	0.87	1.69	1.27	3.08	0.48	0.60	2.06	2.09	1.11	4.90

**Marital status**												
Single ^†^	1.00	1.00	1.00	1.00	1.00	1.00	1.00	1.00	1.00	1.00	1.00	1.00
Married/living together	1.04	1.16	0.97	0.71	0.83	0.86	0.70	1.19	1.51	1.28	0.84	0.53
No longer married	1.07	1.04	2.06	0.66	0.72	0.56	0.47	0.38	1.07	0.68	0.71	0.50

**Social support**												
Low ^†^	1.00	1.00	1.00	1.00	1.00	1.00	1.00	1.00	1.00	1.00	1.00	1.00
Medium	0.55	0.48	1.58	2.42	2.60	4.06	1.28	2.10	1.72	1.84	1.86	4.06
High	0.62	0.60	1.01	1.30	**2.98**	**4.89**	0.95	2.11	1.65	1.88	2.06	3.67

**Education**												
No qualifications ^†^	1.00	1.00	1.00	1.00	1.00	1.00	1.00	1.00	1.00	1.00	1.00	1.00
Secondary school	0.87	1.29	2.17	4.53	1.03	1.27	0.46	0.41	0.83	0.69	1.36	0.98
Higher education	1.18	2.10	2.56	5.19	1.35	2.09	0.65	0.61	0.94	0.80	1.34	1.09

**Housing**												
Owned/mortgaged ^†^	1.00	1.00	1.00	1.00	1.00	1.00	1.00	1.00	1.00	1.00	1.00	1.00
Privately rented and other	0.99	1.21	1.23	2.40	0.77	0.62	1.42	0.94	1.13	1.41	0.72	0.65
Council/housing assoc.	0.80	0.98	0.89	1.21	0.81	0.89	0.95	0.52	0.94	1.19	0.50	0.31

**Employment**												
Full-time ^†^	1.00	1.00	1.00	1.00	1.00	1.00	1.00	1.00	1.00	1.00	1.00	1.00
Part-time	1.06	1.25	1.03	0.98	1.74	1.87	0.83	0.89	1.15	0.82	1.16	1.43
Self-employed	0.81	0.69	0.50	0.36	1.90	1.19	0.69	0.55	0.84	0.57	0.98	1.11
Not working due to illness	1.53	0.99	0.95	0.10	1.32	0.69	2.08	2.52	0.66	0.46	1.88	1.13
Others not in employment	1.09	1.68	0.76	1.03	1.20	1.18	1.69	2.69	0.86	0.83	0.98	1.24

**Income**												
<£15,000 ^†^	1.00	1.00	1.00	1.00	1.00	1.00	1.00	1.00	1.00	1.00	1.00	1.00
£15,000-29,999	1.17	1.56	0.69	0.48	0.96	0.65	0.67	1.30	1.66	1.09	1.07	1.15
£30,000-49,000	1.01	1.20	0.74	0.66	1.08	0.80	0.78	1.28	1.41	0.82	0.76	0.83
£50,000+	1.01	1.00	0.80	0.67	0.55	0.36	0.59	0.67	1.38	0.77	1.01	1.11

**Ethnicity**												
White ^†^	1.00	1.00	1.00	1.00	1.00	1.00	1.00	1.00	1.00	1.00	1.00	1.00
Other	2.66	**5.61**	6.41	3.18	1.30	1.62	**8.00**	**26.92**	0.71	0.88	1.13	2.97

**Smoking**												
Never ^†^	1.00	1.00	1.00	1.00	1.00	1.00	1.00	1.00	1.00	1.00	1.00	1.00
Ex-smoker	1.09	1.15	1.11	1.23	0.98	0.76	0.84	0.93	0.88	0.93	1.01	0.95
Current smoker	0.99	0.90	0.56	0.53	1.16	0.92	0.96	0.61	0.61	0.95	1.10	1.40

**Chronic condition**												
No ^†^	1.00	1.00	1.00	1.00	1.00	1.00	1.00	1.00	1.00	1.00	1.00	1.00
Yes	0.98	0.87	0.93	1.17	0.94	0.94	1.21	0.91	0.91	0.88	0.85	0.73

**Severity of symptom**												
Low ^†^	1.00	1.00	1.00	1.00	1.00	1.00	1.00	1.00	1.00	1.00	1.00	1.00
High	1.66	1.05	6.95	4.48	**3.61**	2.85	**4.19**	2.80	1.65	1.08	**3.09**	**3.21**

**Duration of symptom**												
Short ^†^	1.00	1.00	1.00	1.00	1.00	1.00	1.00	1.00	1.00	1.00	1.00	1.00
Medium	**2.71**	**3.10**	2.89	**2.71**	1.63	1.81	1.72	2.22	1.38	1.24	**2.62**	2.04
Long	**1.88**	1.99	4.08	5.51	**2.00**	1.77	1.77	1.52	0.80	0.57	**1.85**	1.80

**Interference with life**												
Low ^†^	1.00	1.00	1.00	1.00	1.00	1.00	1.00	1.00	1.00	1.00	1.00	1.00
High	**2.08**	**2.30**	**8.20**	**6.93**	**4.75**	2.01	**2.51**	2.28	1.98	2.24	**3.49**	1.77

**Recent experience**												
No ^†^	1.00	1.00	1.00	1.00	1.00	1.00	1.00	1.00	1.00	1.00	1.00	1.00
Yes	0.80	0.64	0.49	**0.25**	0.53	0.47	0.93	0.59	1.37	1.15	0.66	1.21

^† ^Referent group

UOR - unadjusted odds ratio

AOR - adjusted odds ratio (adjusted for all variables, except when the variable itself was being examined

Figures highlighted in bold are significant at 1% level (p < 0.01) (99% CIs are not presented to simplify presentation)

Table 4 shows the factors associated with consulting a primary care health professional for the same six symptoms. Few participant characteristics were significantly associated with the use of primary care health professionals. Women, those not working due to illness, and those with a chronic condition were more likely to consult for some symptoms than those in the reference group for each of these characteristics, while those with a higher annual household income were less likely to consult. After adjustment, only the relationship with gender remained statistically significant among the personal characteristics. Symptom characteristics were more often associated with the use of primary care health professionals. While some of the significant associations were lost after adjustment, most of the trends remained.

**Table 4:** Associations between use of primary care professionals for selected symptoms and participant and symptom characteristics (unadjusted and adjusted odds ratios)

Participant and symptom characteristics	Feeling tired/ run down		Cold or flu symptoms		Back pain		Nervousness/ anxiety		Headaches		Joint pain	
	**UOR**	**AOR**	**UOR**	**AOR**	**UOR**	**AOR**	**UOR**	**AOR**	**UOR**	**AOR**	**UOR**	**AOR**

**Gender**												
Men ^†^	1.00	1.00	1.00	1.00	1.00	1.00	1.00	1.00	1.00	1.00	1.00	1.00
Women	1.71	1.64	2.44	1.93	**2.09**	2.10	1.34	1.75	**2.25**	**6.14**	**1.96**	**2.59**

**Age**												
18-24 ^†^	1.00	1.00	1.00	1.00	1.00	1.00	1.00	1.00	1.00	1.00	1.00	1.00
25-34 yrs	0.72	0.37	0.58	0.14	0.86	1.19	0.76	0.71	0.55	1.41	1.23	1.01
35-44 yrs	1.26	1.45	0.56	0.23	2.29	3.47	1.01	1.89	1.47	4.55	2.07	2.17
45-54 yrs	1.54	1.10	0.85	0.31	1.77	3.61	1.63	3.08	1.61	2.77	2.40	2.22
55-60 yrs	1.23	0.60	0.85	0.27	2.58	4.92	1.23	5.54	2.32	4.63	3.12	3.57

**Marital status**												
Single ^†^	1.00	1.00	1.00	1.00	1.00	1.00	1.00	1.00	1.00	1.00	1.00	1.00
Married/living together	1.09	1.38	0.95	1.29	1.18	0.92	0.90	1.74	1.30	1.71	1.26	0.90
No longer married	2.18	1.58	2.89	0.62	1.62	0.48	1.53	0.84	1.29	0.31	1.63	0.57

**Social support**												
Low ^†^	1.00	1.00	1.00	1.00	1.00	1.00	1.00	1.00	1.00	1.00	1.00	1.00
Medium	0.51	0.31	3.50	3.56	0.95	2.88	0.48	0.22	1.33	2.20	0.99	3.07
High	0.48	0.49	2.01	1.39	1.44	6.02	0.33	0.22	1.17	0.95	1.32	3.78

**Education**												
No qualifications ^†^	1.00	1.00	1.00	1.00	1.00	1.00	1.00	1.00	1.00	1.00	1.00	1.00
Secondary school	0.79	2.61	1.93	14.09	0.66	0.95	0.48	1.16	0.40	0.18	0.64	1.02
Higher education	0.40	1.65	1.09	15.74	0.78	2.54	0.36	0.83	0.36	0.10	0.67	1.49

**Housing**												
Owned/mortgaged ^†^	1.00	1.00	1.00	1.00	1.00	1.00	1.00	1.00	1.00	1.00	1.00	1.00
Privately rented and other	1.43	0.60	1.50	1.00	0.87	1.02	1.50	1.30	1.28	2.08	0.57	0.63
Council/housing assoc.	1.39	0.74	1.18	0.28	1.09	1.19	1.19	0.27	1.33	0.06	1.15	0.50

**Employment**												
Full-time ^†^	1.00	1.00	1.00	1.00	1.00	1.00	1.00	1.00	1.00	1.00	1.00	1.00
Part-time	1.88	1.18	1.06	0.89	1.78	0.98	1.27	1.15	1.14	0.50	1.51	0.90
Self-employed	0.60	0.51	0.71	0.43	1.35	1.16	0.37	0.43	0.50	0.16	0.78	0.69
Not working due to illness	**6.52**	2.57	2.76	0.66	**9.46**	2.50	**5.00**	5.54	**5.31**	4.09	**14.68**	3.63
Others not in employment	1.03	0.79	1.07	0.45	1.44	0.54	1.17	1.63	0.96	0.24	1.15	0.58

**Household income**												
<£15,000 ^†^	1.00	1.00	1.00	1.00	1.00	1.00	1.00	1.00	1.00	1.00	1.00	1.00
£15,000-29,999	0.76	0.75	0.93	3.84	0.69	0.58	**0.35**	0.55	0.93	0.62	0.83	0.78
£30,000-49,000	0.47	0.40	0.50	0.92	0.53	0.51	0.52	0.80	0.71	0.40	**0.45**	0.70
£50,000+	0.44	0.80	0.60	1.15	**0.25**	0.20	0.51	1.73	0.38	0.40	0.47	0.62

**Ethnicity**												
White ^†^	1.00	1.00	1.00	1.00	1.00	1.00	1.00	1.00	1.00	1.00	1.00	1.00
Other	0.86	2.48	9.49	84.62	1.19	0.67	1.82	6.46	2.15	5.47	1.83	2.91

**Smoking**												
Never ^†^	1.00	1.00	1.00	1.00	1.00	1.00	1.00	1.00	1.00	1.00	1.00	1.00
Ex-smoker	1.69	1.59	1.20	0.96	1.50	0.71	1.77	1.30	1.16	0.23	1.56	1.24
Current smoker	1.62	0.98	0.44	0.55	0.94	0.64	2.00	1.91	0.82	0.50	1.14	0.82

**Chronic condition**												
No ^†^	1.00	1.00	1.00	1.00	1.00	1.00	1.00	1.00	1.00	1.00	1.00	1.00
Yes	**3.11**	2.31	1.06	1.94	**1.76**	1.41	**5.05**	2.17	1.50	1.10	**2.11**	1.36

**Severity of symptom**												
Low^†^	1.00	1.00	1.00	1.00	1.00	1.00	1.00	1.00	1.00	1.00	1.00	1.00
High	**5.97**	2.64	19.78	2.50	**11.70**	**4.33**	**12.07**	**8.47**	**4.55**	4.24	**7.68**	**5.09**

**Duration of symptom**												
Short^†^	1.00	1.00	1.00	1.00	1.00	1.00	1.00	1.00	1.00	1.00	1.00	1.00
Medium	2.03	2.06	3.98	3.03	**3.50**	2.99	1.76	0.97	**4.61**	6.49	**2.67**	1.96
Long	**4.66**	2.66	6.96	12.23	**7.06**	**3.75**	**5.18**	2.09	1.65	0.19	**3.14**	**2.23**

**Interference with life**												
Low^†^	1.00	1.00	1.00	1.00	1.00	1.00	1.00	1.00	1.00	1.00	1.00	1.00
High	**5.58**	**2.86**	**22.83**	**19.42**	**10.55**	**3.63**	**6.03**	**4.38**	**11.07**	**15.83**	**8.54**	**3.13**

**Recent experience**												
No^†^	1.00	1.00	1.00	1.00	1.00	1.00	1.00	1.00	1.00	1.00	1.00	1.00
Yes	0.65	0.27	0.50	0.15	0.65	0.83	0.63	0.12	0.94	0.52	0.70	1.04

^† ^Referent group

UOR - unadjusted odds ratio

AOR - adjusted odds ratio (adjusted for all variables, except when the variable itself was being examined)

Figures highlighted in bold are significant at 1% level (p < 0.01) (99% CIs are not presented to simplify presentation)

Similar patterns of associations were seen for other symptoms examined (data not shown) i.e. with few participant characteristics and more symptom characteristics associated with the actions taken.

## Discussion

### Summary of main findings

This study has shown that most symptom episodes are not currently presented to healthcare services in the UK and remain in the submerged portion of the symptom iceberg. Doing nothing about the symptom was the most common response, followed by use of an OTC medicine. The GP was the most common health professional consulted, while use of other primary care health professionals was very low. Actions taken for individual symptoms varied substantially although a number of broad management patterns were evident. The number and characteristics of symptoms experienced were more commonly associated with the actions taken than participant characteristics.

### Strengths and limitations of the study

This is the first UK-wide population-based study to examine the actions people take to manage their symptoms since the changes in primary care were introduced. It investigated a wide range of symptoms, including physical and psychological symptoms and symptoms ranging from those usually indicative of minor illness to those which could be indicative of serious conditions. The response rate was low, an increasingly common problem in epidemiological research [14,15]. In addition to the usual reasons for non-response, our questionnaire: a) was a general health questionnaire not targeted at people with a specific condition so some people may have felt the questionnaire was not relevant to them; b) was relatively long and quite complex to complete; c) asked for a lot of detail on health and demographics that some individuals may not have been keen to provide; d) was sent only to a working age population, thereby excluding older people known to be more likely to respond; and e) was sent to a number of areas of high deprivation known to be associated with poorer response rates. Despite the low response rate, the relatively large sample size and recruitment of practices from a wide variety of geographical and socio-economic areas ensured that most demographic and socio-economic groups (with the exception of non-whites) were well represented, thus allowing important sub-group analysis and providing a good level of generalisability for the working age population of the UK. The study found symptom prevalence rates comparable with other studies and reported similar proportions of service use as other studies, suggesting low response bias.

As this was a cross-sectional study the findings are a single snapshot in time of symptom experience and actions taken to manage them. The study will therefore have captured new symptoms, recurrent symptoms and symptoms of chronic complaints. It is important to remember that people may have previously consulted or subsequently consulted health care professionals about their symptoms outwith the time period examined in this study. People's previous experience of the reported symptom and previous actions taken to manage this symptom are likely to have influenced the actions taken for this symptom episode.

All data were self-reported and so were susceptible to recall bias. Efforts were made to minimise this by asking about the last two weeks, however some recall bias may still have occurred. Our findings may also have been susceptible to retrospective bias (people exaggerating the characteristics of symptoms in an attempt to justify the use of services or medicines). We do not believe this was a particular problem in our study since people were asked about symptom characteristics prior to actions taken.

We adjusted for a wide range of demographic, socio-economic and symptom characteristics. However, there may have been a number of potentially important characteristics that were not measured (e.g. lifestyle factors), and some residual confounding may have occurred as a result. Finally, the small numbers of some symptoms and actions taken means that the study lacked statistical power to detect differences between some groups.

### Comparison with existing literature

There have been relatively few community-based studies investigating the publics' responses to a range of different symptoms. Many have taken place outwith the UK and most were conducted 20-50 years ago. There have been no community based studies in the UK since the recent primary care changes were introduced. As a result there is no current UK information with which to directly compare our findings. In addition, differences in the populations studied, symptoms enquired about, timeframe over which symptoms are examined, and actions investigated in previous studies make comparisons across studies difficult.

In our study, nearly half of all symptoms resulted in respondents taking no action over the two week period. This finding is broadly consistent with some studies [8,16], while others have reported a lower proportion of symptoms leading to no action and a higher proportion leading to self-care [10-13,17,18]. These studies have tended to use a broader definition of self-care (which sometimes included doing nothing), and asked about a wider range of lay-care strategies (such as rest, exercise, home remedies, diet changes) than we did.

Our finding that 12% of symptoms resulted in a consultation with a primary care health professional is consistent with previous estimates of 5-34% [8,13,17,19]. Previous estimates of the proportion of symptoms presenting specifically to a GP have also been similar [18,20,21]. There have been no UK community studies investigating the use of the wider primary care team in response to symptoms. This study therefore provides important information about the low use of other primary care health professionals for managing symptoms.

A number of previous studies have shown that certain population groups are more likely to consult a GP for their symptoms than others including women [1,11,16,22-25], older age groups [11,16,22], those not employed [11,22,23], those in lower social classes [22], and those with a higher number of symptoms or chronic conditions [23,26-28]. Few studies have examined the factors associated with the use of other actions, although some have found that certain groups are more likely to use self-care [10,11,13]. Although we found some evidence of demographic and socio-economic factors associated with actions taken, these associations were not as strong as previous studies have suggested. The associations varied considerably by the action taken, as well as by the individual symptom examined. For example, when all symptoms were combined we did not find that women were more likely to consult a GP than men. However, we did find that women were more likely to have consulted a primary care health professional for *headaches *and *joint pain*. A previous UK study also reported that women are no more likely to consult the GP than men for most symptoms [29].

In this study, symptom characteristics were more strongly and consistently associated with actions taken. Few previous studies have looked at symptom characteristics when examining responses to symptoms. Those which have, consistently report that symptom characteristics are more strongly related to actions taken than demographic and socio-economic characteristics [11,13,16,18,28]. This suggests that apparent associations with demographic and socio-economic characteristics seen in some studies (which were not able to consider symptom characteristics) may be accounted for, at least in part, by the symptom characteristics themselves.

### Conclusions and implications for future research

Our results provide a detailed current picture of 25 symptom icebergs in the UK. While use of lay-care for minor ailments was widespread, use of the primary care team other than the GP was very low. Further research is required to examine the public's knowledge of and opinions on the services offered by different members of the primary care team and investigate why certain services do not appear to be being used. Such information will be crucial to inform the future development of primary care in the UK.

## Funding

The study was funded by a Wellcome Trust Research Career Development Fellowship for Dr Alison M Elliott (grant reference number: 078176/Z/05/Z). The funders had no role in study design, in the analysis and interpretation of data, in the writing of the report, or in the decision to submit the article for publication.

## Ethical approval

Ethical Approval for the study was granted by the Fife and Forth Valley Research Ethics Committee as part of the NHS Multi-Centre Research Ethics Committee for Scotland (Ref. 06/S0501/71).

## Competing interests

All authors declare that they have no financial or non-financial interests that may be relevant to the submitted work and therefore have nothing to declare.

## Authors' contributions to the paper

AME and PCH had the original idea for the research and developed the proposal. AM and AME conducted the data collection and analysis. AME wrote the first and subsequent drafts of the paper. All authors contributed to the scientific development of the paper, commented on successive drafts and agreed to the final manuscript.

## Pre-publication history

The pre-publication history for this paper can be accessed here:

http://www.biomedcentral.com/1471-2296/12/16/prepub
